# Phenotypic Heterogeneity of Breast Cancer Stem Cells

**DOI:** 10.1155/2011/135039

**Published:** 2011-01-17

**Authors:** Aurelio Lorico, Germana Rappa

**Affiliations:** Division of Translational Science, Nevada Cancer Institute, One Breakthrough Way, Las Vegas, NV 89135, USA

## Abstract

Many types of tumors are organized in a hierarchy of heterogeneous cell populations, with only a small proportion of cancer stem cells (CSCs) capable of sustaining tumor formation and growth, giving rise to differentiated cells, which form the bulk of the tumor. Proof of the existence of CSC comes from clinical experience with germ-cell cancers, where the elimination of a subset of undifferentiated cells can cure patients (Horwich et al., 2006), and from the study of leukemic cells (Bonnet and Dick, 1997; Lapidot et al., 1994; and Yilmaz et al., 2006). The discovery of CSC in leukemias as well as in many solid malignancies, including breast carcinoma (Al-Hajj et al. 2003; Fang et al., 2005; Hemmati et al., 2003; Kim et al., 2005; Lawson et al., 2007; Li et al., 2007; Ricci-Vitiani et al., 2007; Singh et al., 2003; and Xin et al., 2005), has suggested a unifying CSC theory of cancer development. The reported general insensitivity of CSC to chemotherapy and radiation treatment (Bao et al., 2006) has suggested that current anticancer drugs, which inhibit bulk replicating cancer cells, may not effectively inhibit CSC. The clinical relevance of targeting CSC-associated genes is supported by several recent studies, including CD44 targeting for treatment of acute myeloid leukemia (Jin et al., 2006), CD24 targeting for treatment of colon and pancreatic cancer (Sagiv et al., 2008), and CD133 targeting for hepatocellular and gastric cancer (Smith et al., 2008). One promising approach is to target CSC survival signaling pathways, where leukemia stem cell research has already made some progress (Mikkola et al., 2010).

## 1. Cancer Stem Cells

In the past few years, a growing body of experimental evidence has been reported in favor of the hypothesis that many types of tumors are organized in a hierarchy of heterogeneous cell populations, with only a small proportion of cancer stem cells (CSCs) capable of sustaining tumor formation and growth, giving rise to differentiated cells, which form the bulk of the tumor. Proof of the existence of CSC comes from clinical experience with germ-cell cancers, where the elimination of a subset of undifferentiated cells can cure patients [1], and from the study of leukemic cells [2–4]. The discovery of CSC in leukemias as well as in many solid malignancies, including breast carcinoma [5–13], has suggested a unifying CSC theory of cancer development. The reported general insensitivity of CSC to chemotherapy and radiation treatment [14] has suggested that current anticancer drugs, which are developed extensively based on their activity to inhibit bulk replicating cancer cells, may not effectively inhibit CSC, and that targeting CSC will be helpful in eradicating tumors more efficiently. The clinical relevance of targeting CSC-associated genes is supported by several recent studies, including CD44 targeting for treatment of acute myeloid leukemia [15], CD24 targeting for treatment of colon and pancreatic cancer [16], and CD133 targeting for hepatocellular and gastric cancer [17]. One promising approach is to target CSC survival signaling pathways, where leukemia stem cell research has already made some progress [18].

## 2. Breast Cancer Stem Cells

Breast cancer, a complex and heterogeneous disease, is the leading cause of cancer death in women. More than a million new cases are diagnosed every year worldwide [19]. Despite combined treatment with surgery, radiotherapy, and anticancer drugs, many breast cancer patients will ultimately develop metastatic disease, at present incurable. While many studies have attempted to demonstrate the presence of breast CSC (BCSC) based on cell surface marker profiles, consensus on their phenotypic characterization is still missing. In light of recent experimental evidence, the idea of a universal marker or combination of markers able to identify and isolate BCSC from all breast cancers seems unrealistic. This is not surprising, because breast cancer is not a single disease; it is comprised of various histological subtypes, with variable clinical presentations and different underlying molecular signatures. On the basis of global gene expression profiling, breast cancer has been divided into five major molecular subtypes: luminal A, luminal B, HER2+, basal-like, and normal breastlike [20–22]. Each subtype is associated with a peculiar natural history and treatment responsiveness. Thus, the prognosis of patients with basal-like tumors is worse than for patient with luminal A tumors [22, 23]. In addition to intertumor heterogeneity, there is also a high degree of intratumor diversity. Specifically, a single tumor at any given time can contain tumor cell populations with distinct molecular profiles and biological properties. Intratumor diversity has been reported as early as at the stage of ductal carcinoma in situ [24, 25]. Heterogeneity of CSC populations has been demonstrated in other types of tumors, such as glioblastoma, where different CSC subpopulations have been described [26, 27]. Park et al. [23], based on their recent immunohistochemical analyses of 12 markers in almost 400 ductal breast cancers, concluded that the frequency of breast cancer cells positive for stem cell-like and more differentiated markers varies according to tumor subtype and histologic stage. A concise review of the literature for the most studied BCSC markers follows.

## 3. Original CD44^+^/CD24^−/low^ BCSC Phenotype

A CD44^+^/CD24^−/low^ subpopulation of CSC was originally identified from Al-Haji et al. [5], using cells from metastatic pleural effusions of breast carcinoma patients. Their presence has subsequently been confirmed in additional studies, especially in the MCF7 cell line [28]. Following removal of nonepithelial cells, cells with the CD44^+^CD24^−/low^ phenotype were highly enriched in their ability to initiate tumors compared with unsorted cells. Further enrichment was possible by additionally sorting the cells for expression of the ESA (epithelial cell adhesion molecule) antigen. CD44^+^/CD24^−/low^ were also able to serially propagate the tumors in mice, demonstrating capacity for self-renewal. CD24 is a heavily glycosylated, mucin-type protein linked to the cell membrane via glycosyl-phosphatidylinositol [29]. Since it can bind P-selectin, a lectin expressed by vascular endothelium and platelets, it has been suggested to play an important role in the metastatic process [30, 31]. CD44 is a transmembrane glycoprotein, present in several isoforms, that normally takes part in cell-cell and cell-matrix adhesion interactions, and cell migration. CD44 binds hyaluronic acid as well as collagen, fibronectin, laminin, and chondroitin sulfate, important components of the extracellular matrix, as well as the cytokine osteopontin [32]. Many cancer cell types as well as their metastases express high levels of CD44 and/or CD44 variants. Since the blockage of CD44-ligand interaction inhibits local tumor growth and metastatic spread, CD44 may confer a growth advantage to breast cancer cells. The initial reports that only the CD44^+^CD24^−/low^ subpopulation of human breast cancer cells contains BCSC have been challenged by subsequent studies [33, 34]. Honeth et al. [34] detected a CD44^+^CD24^−/low^ subpopulation in only 31% of 240 human breast cancer samples analyzed, with a strong association with the basal-like phenotype. Creighton et al. [35] reported that a gene expression signature common to both CD44^+^/CD24^−/low^ and mammosphere-forming cells was mainly present in breast cancer of the recently identified claudin-low molecular subtype, which is characterized by expression of many epithelial-mesenchymal transition- (EMT-) associated genes. In addition, contrasting results have been reported by different groups in regard to the invasiveness of CD44^+^CD24^+^ compared with CD44^+^CD24^−/low^ cells [30, 36–38]. We [39] and other groups [33, 34] have found that CD24 is not a consistent breast cancer stem cell marker. In particular, in a human breast carcinoma model originated from bone marrow micrometastases of a breast cancer patient [40], we have recently shown that the s.c growth of CD24^+^ and CD24^−^ sorted breast cancer cell subpopulations and their single-cell clones resulted in similar take and growth rates [39]. Single cell-sorted CD24^−^ and CD24^high^ MA-11 gave rise *in vitro* to cell populations with heterogeneous CD24 expression. Also, all tumor xenografts derived from CD24^+^ and CD24^−^ cells expressed CD24 on their cell surface *in vivo* [39]. The rapid up- and downregulation of putative stem cell markers is not novel; Monzani et al. [41] have recently shown that after injecting CD133^+^ melanoma cells in NOD-SCID mice, most of the tumors became CD133 negative. Furthermore, growing these cells *in vitro* after few passages, they re-expressed CD133. That CD24 is rapidly and transiently downregulated under certain culture conditions reconciles the apparent discrepancy of the promalignant and proinvasiveness role of CD24 with the CD24^−/low^ phenotype of breast CSC [5, 28, 30, 33, 34, 37, 38]. Interestingly, CD24 silencing did not change tumorigenicity, suggesting that the level of expression of CD24 is associated with but does not contribute to tumorigenicity [39]. These findings, together with the widespread expression of CD44, strongly suggest that the CD44^+^CD24^−/low^ phenotype is not sufficient to characterize BCSC. 

## 4. Mammosphere Formation

Based on the mammosphere-forming assay in serum-free medium on nonadherent plastic used for culture of normal mammary epithelial cells, Ponti and colleagues employed a similar approach to derive mammospheres from human breast cancers [28] (Table 1). They found in the malignant mammospheres the same CD44^+^CD24^−^ phenotype reported by Al-Hajj et al. [5], and the capacity to differentiate to both luminal and basal/myoepithelial lineages. Fournier et al. [42, 52] showed the importance of 3D cultures for the generation of breast cancer signatures. Rappa et al. [39, 53] found an increased expression of surface markers associated with the stem cell phenotype and of oncogenes in cell lines and clones cultured as spheroids versus adherent cultures; also, spheroid-forming cells displayed increased tumorigenicity and an altered pattern of chemosensitivity. MAPK, Notch, and Wnt-associated genes, along with the BCSC marker, aldehyde dehydrogenase, were found overexpressed in mammospheres from breast cancer cell lines. 

## 5. Side Population

The ability to exclude the Hoescht 33342 fluorescent dye from the intracellular compartment, originally developed by the Goodell lab to isolate a “side population” (SP) of hematopoietic cells highly enriched in haematopoietic stem cells [54], results from the expression of ATP-binding cassette (ABC) transporters. A similar SP, enriched in cells with the ability to initiate tumors in immune deficient mice, has been identified in breast cancer cell lines [43, 44]. Also, enrichment for the progenitor cell-containing SP after irradiation was observed in breast cancer cell lines [55]. However, unresolved issues with potential toxicity of Hoechst 33342 to non-SP cells hinder the further application of this functional assay to the identification of BCSC subpopulations. 

## 6. Aldehyde Dehydrogenase

Another candidate marker for a breast CSC phenotype is aldehyde dehydrogenase (ALDH). ALDHs are a family of enzymes involved in the detoxification of a wide variety of aldehydes to their corresponding weak carboxylic acids, including xenobiotic aldehydes, such as cyclophosphamide [56]. Since there are 19 ALDH genes in humans, organized into 11 groups, with functional overlap, a functional enzymatic assay, rather than immunohistochemistry methods, is generally used to identify ALDH+ cells, employing the commercial reagent ALDEFLUOR (STEMCELL Technologies Inc, Vancouver, BC, Canada). The ALDEFLUOR substrate, BODIPY aminoacetaldehyde (BAAA), is converted by ALDH in the cells into a fluorescent molecule, that accumulates in cells in the presence of efflux inhibitors, allowing cells with high ALDH activity to be easily identified. Ginestier et al. [45] reported that only 20% to 25% of breast carcinomas express ALDH. Of these, on average, 5% of the cells are positive for ALDH. A minor overlap (1%) between the ALDEFLUOR-positive population and the CD44^+^/CD24^−/low^ subpopulation was observed in that study. However, as few as 20 cells expressing both BCSC phenotypes were required to generate a tumor in immunodeficient mice. In the same study, ALDH− cells were not tumorigenic up to 50,000 cells, and ALDH+ tumors were associated with high histological grade, ERBB2 overexpression, absence of estrogen and progesterone receptor expression, and poor clinical outcome, based on overall survival. These observations led the authors to propose that ALDH expression in a subset of tumors may reflect transformation of ALDH+ stem or early progenitor cells in these tumors. By contrast, ALDH− tumors may be generated by the transformation of ALDH− progenitor cells. In apparent contrast with the relatively low percentage of ALDH+ breast cancers *in vivo*, Charafe-Jauffret et al. [46] reported that 23 of 33 cell lines derived from normal and malignant mammary tissue contained an ALDEFLUOR+ population that displayed stem cell properties *in vitro* and in NOD/SCID xenografts. Also, another study from the same group demonstrated that ALDH1 expression can be an independent prognostic factor for predicting metastases in inflammatory breast cancer and that CSCs have the ability to reconstitute the heterogeneity of the primary tumor at the metastatic site [57].

## 7. Prominin-1 (CD133)

Recent data from several laboratories suggest that CD133-positivity identifies a subgroup of breast CSC [47–49]. CD133, named Prominin-1 for its prominent location on the protrusion of cell membranes, is the first identified gene in a class of novel pentaspan transmembrane glycoproteins [58, 59]. It defines a broad population of somatic stem and progenitor cells, including those derived from the hematopoietic and nervous system [60]. In addition, it has been found to be elevated in peripheral blood of patients with metastatic cancer [61]. CD133 is much more restricted in expression compared with other CSC markers such as CD44 and ALDH, which are more universally expressed in normal as well as cancer cells. A subpopulation of CD133^+^ CSC has been identified in colon carcinoma [11] and glioblastoma [12]. Although CD133 is considered the most important CSC marker identified so far, very little is known about its physiological function(s), except in the eye, where, together with protocadherin 21, a photoreceptor-specific cadherin, and with actin filaments, it forms a complex involved in photoreceptor disk morphogenesis [62]. Also, current knowledge about the regulatory mechanisms and the interaction of CD133 with other cellular proteins and biochemical pathways is very scarce. We have reported [63] in malignant melanoma that shRNA-mediated downregulation had profound effects on human CD133-expressing cancer cells; *in vitro* CD133 knockdown slowed cell growth, reduced cell motility, and decreased the formation of spheroids under stem cell-like growth conditions; *in vivo* the downregulation of CD133 severely reduced the capacity of the cells to metastasize, particularly to the spinal cord [63]. Successful immunotoxin targeting of CD133 in hepatocellular and gastric cancer xenografts has also been reported [17]. These data suggest that CD133, in addition to its role as a CSC marker, is an important cancer therapeutic target. Expression of CD133 has recently been reported in 22 out of 25 cases of inflammatory breast cancer (IBC), a particularly lethal form of breast cancer characterized by exaggerated lymphovascular invasion [47]. CD133 expression was also detected in BCSC-enriched spheroids of the MARY-X xenograft model of IBC [47]. Interestingly, MARY-X spheroids expressed a BCSC profile characterized as CD44^+^/CD24^−/low^, ALDH^+^, and CD133^+^ [47]. Also, in BRCA1-associated breast cancer cell lines, CD133^+^ sorted cells harbor CSC properties such as a greater colony-forming efficiency, higher proliferative output, and greater ability to form tumors in NOD/SCID mice [48]. In addition, basal-like breast carcinoma cells from patients and stem/progenitor cells of mammospheres isolated from ductal breast carcinoma express high levels of CD133 [49]. 

## 8. Integrins

Mouse mammary stem cells have recently been identified by employing the integrins CD29 (*β*1) and CD49f (*α*6) in combination with CD24 [64, 65]. Based on the hypothesis that markers used for normal mammary stem cells could also work for the isolation of mammary CSC, Vassilopolous et al. [50] used CD24/CD29 or CD24/CD49f to identify a subpopulation of mammary tumor cells. In addition, a mammary progenitor cell population has been shown to express high levels of integrin CD61 (*β*3), which is only marginally expressed in normal mammary epithelia [66]. Employing three different mouse models of mammary tumorigenesis, Vaillant et al. [51] found that in two of them (MMTV-wnt-1, and p53^+/−^), CD61 identified a subpopulation that was highly enriched for tumorigenic capability relative to the CD61^−^ subset. 

## 9. Conclusions

It is conceivable that breast cancer heterogeneity derives, at least in part, from the existence of distinct BCSC populations. Available markers should be further tested in combination; additional markers, or specific gene signatures, are definitely needed to define, and possibly target, BCSC populations of the different breast cancer subtypes. However, the possibility of (i) marker downregulation/silencing; (ii) generation of marker-negative from marker-positive BCSC cells; (iii) coexistence of different BCSC subpopulations in the same tumor or at different metastatic sites should be considered before designing novel anti-BCSC strategies.

## Figures and Tables

**Table 1:** 

Breast cancer stem cell markers	References
CD44^+^CD24^−/low^	Al-Haji et al. [5]; Ponti et al. [28]; Honeth et al. [34]
Mammosphere-forming ability	Ponti et al. [28]; Fournier and Martin [42]; Rappa and Lorico [39]
Hoechst 33342 side population	Hirschmann-Jax et al. [43]; Patrawala et al. [44]
Aldehyde dehydrogenase	Ginestier et al. [45]; Charafe-Jauffret et al. [46]
CD133	Xiao et al. [47]; Wright et al. [48]; Storci et al. [49]
Integrins	Vassilopolous et al. [50]; Vaillant et al. [51]
